# Ku70 Alleviates Neurodegeneration in Drosophila Models of Huntington's Disease

**DOI:** 10.1371/journal.pone.0027408

**Published:** 2011-11-07

**Authors:** Takuya Tamura, Masaki Sone, Takeshi Iwatsubo, Kazuhiko Tagawa, Erich E. Wanker, Hitoshi Okazawa

**Affiliations:** 1 Department of Neuropathology, Medical Research Institute, Tokyo Medical and Dental University, Tokyo, Japan; 2 Department of Biomolecular Science, Faculty of Science, Toho University, Funabashi, Japan; 3 Department of Neuropathology, Graduate School of Medicine, University of Tokyo, Tokyo, Japan; 4 Department of Neurogenetics, Max-Delbrück Center for Molecular Medicine, Berlin, Germany; 5 CREST, Japan Science and Technology Agency, Kawaguchi, Japan; VIB & Katholieke Universiteit Leuven, Belgium

## Abstract

DNA damage accumulates in genome DNA during the long life of neurons, thus DNA damage repair is indispensable to keep normal functions of neurons. We previously reported that Ku70, a critical molecule for DNA double strand break (DSB) repair, is involved in the pathology of Huntington's disease (HD). Mutant huntingtin (Htt) impaired Ku70 function via direct interaction, and Ku70 supplementation recovered phenotypes of a mouse HD model. In this study, we generate multiple Drosophila HD models that express mutant huntingtin (Htt) in eye or motor neuron by different drivers and show various phenotypes. In such fly models, Ku70 co-expression recovers lifespan, locomotive activity and eye degeneration. In contrast, Ku70 reduction by heterozygous null mutation or siRNA-mediated knock down accelerates lifespan shortening and locomotion disability. These results collectively support that Ku70 is a critical mediator of the HD pathology and a candidate therapeutic target in HD.

## Introduction

DNA damage repair was recently shown to be a critical component of the polyQ disease pathology, in addition to previously known pathologies including transcription [1]–[4], splicing [5], protein degradation [6]–[8], autophagy [9]–[11], synapse transmission [12]–[14], endocytosis [15], [16], mitochondrial membrane stability [17], [18], energy metabolism [19]–[21] and oxidative stress response [22]–[24].

The first implication of DNA damage repair in the HD pathology came from the age-dependent CAG repeat expansion of the *HD* gene. Oxoguanine glycosylase 1 (OGG1), a critical enzyme for single base excision repair, enhances the age-dependent CAG repeat instability [25], suggesting that genome DNA including the *HD* gene faces continuous DNA damage during life. Another line of evidence for DNA damage repair in HD came from our previous results that mutant Htt impairs HMGB1/2 (high mobility group box 1/2) and Ku70 proteins [26], [27] indispensable for DSB repair [28].

In DNA damage repair, unwinding genomic DNA from histone complex is a crucial process. HMGB1/2 proteins possessing positive- and negative-charge domains play this role through insertion between genomic DNA and histone complex [29]. On the other hand, Ku70/Ku80 heterodimer firstly detects DSBs and assembles at the foci. The heterodimer recruits DNA-PKcs and initiates the non-homologous end joining (NHEJ) type of DSB repair by XRCC4/DNA ligase IV [30]. Therefore, functional impairment of HMGB1/2 or Ku70 by mutant Htt finally increases DSBs and triggers DNA damage signals [26], [27].

In this study, we employ Drosophila genetics to reconfirm involvement of Ku70 in HD pathology and therapeutic application of Ku70 for the fly model. Ku70 overexpression remarkably recovers lifespan, locomotion and eye thickness phenotypes in newly generated multiple Drosophila HD models. Non-sense mutation or RNAi-mediated suppression of Drosophila Ku70 (DmKu70) accelerates mutant Htt-induced phenotypes. These results support involvement of Ku70 in the HD pathology, and strongly suggest availability of Ku70 as a novel therapeutic target.

## Results

### Ku70 rescues mutant Htt-induced eye degeneration in Drosophila

**Figure 1:**
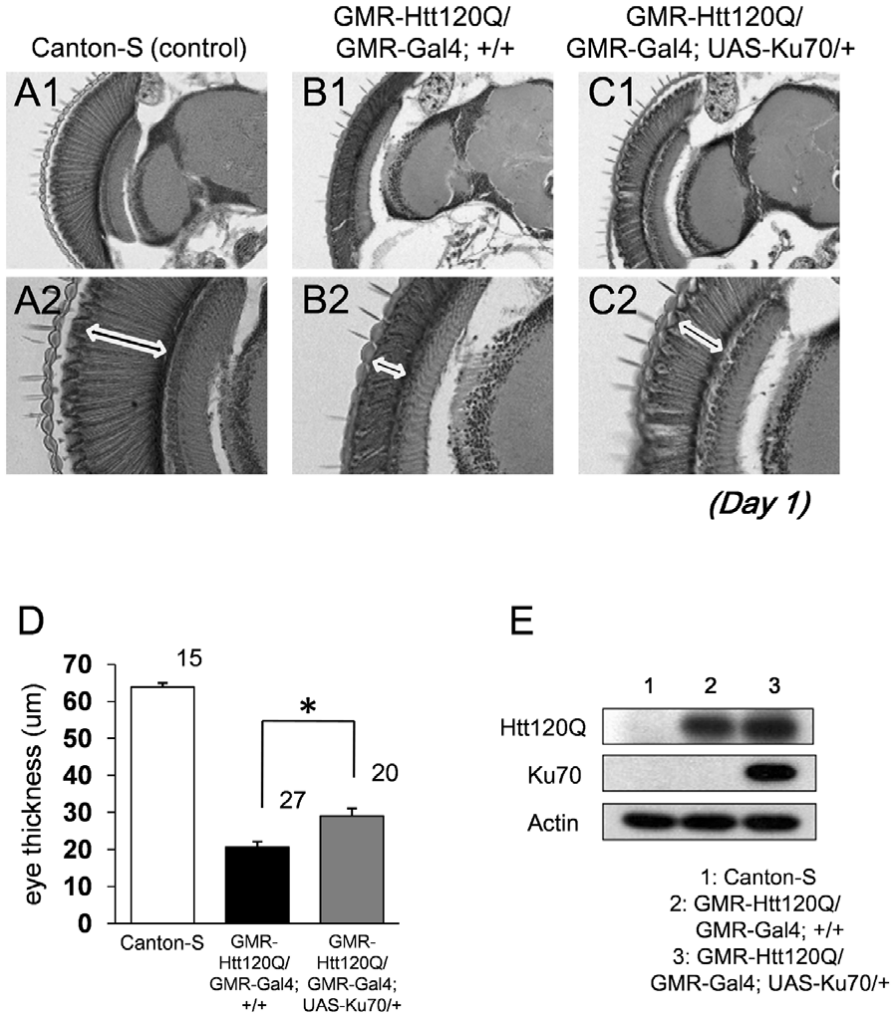
Ku70 reduces eye degeneration of mutant Htt in Drosophila models. (A-C) Axial sections of fly eyes from control Canton-S (A1 and A2), GMR-Htt120Q/+; GMR-Gal4/+ (Htt/+; B1 and B2) and GMR-Htt120Q/+; GMR-Gal4/UAS-mouse Ku70 (Htt/+;Ku70/+; C1 and C2) are shown at a low power (Upper panel) or a high power (Lower panel) magnification. White arrows indicate the retina thickness of each transgenic fly. In triple transgenic flies, co-expression of Ku70 partially repressed eye degeneration. Scale bar, 50 µm. (D) Quantitative analysis of the eye thickness of each transgenic fly. The number of counted flies was indicated above each bar. Mean +/− SE. *: p<0.01, Student's *t*-test. (E) Western blot analysis of the expression of mutant Htt (Htt120Q) and mouse Ku70 (Ku70) in fly eye. All flies tested were female. The genetic background was *w*(CS10) in this figure.

Drosophila has a conserved DSB repair system including Ku70 (DmKu70/IRBP/YPF1/mus309/CG5247), Ku80 (DmKu80/CG18801) and an unidentified DNA-PKcs [31], [32] (http://flybase.bio.indiana.edu). DmKu70's function in DSB is known to be evolutionary conserved [33]. Therefore, we tested the effect of Ku70 on neurodegeneration in Drosophila. Expression of mutant human Htt120Q exon1 peptide (Htt120Q) by GMR-promoter induced degeneration of photoreceptor cells (Figure 1A, 1B). Co-expression of mouse Ku70 by GMR-GAL4/UAS-Ku70 remarkably recovered eye thickness of the Htt120Q transgenic flies (Figure 1C). Quantitative analysis of eye thickness at one day after eclosion confirmed that eye thickness was improved in Htt120Q;Ku70 flies (29.1±2.1 µm) in comparison to Htt120Q transgenic flies (20.6±1.5 µm) (Figure 1D). The expression of mouse Ku70 did not affect the expression level of mutant Htt in the transgenic fly (Figure 1E).

**Figure 2:**
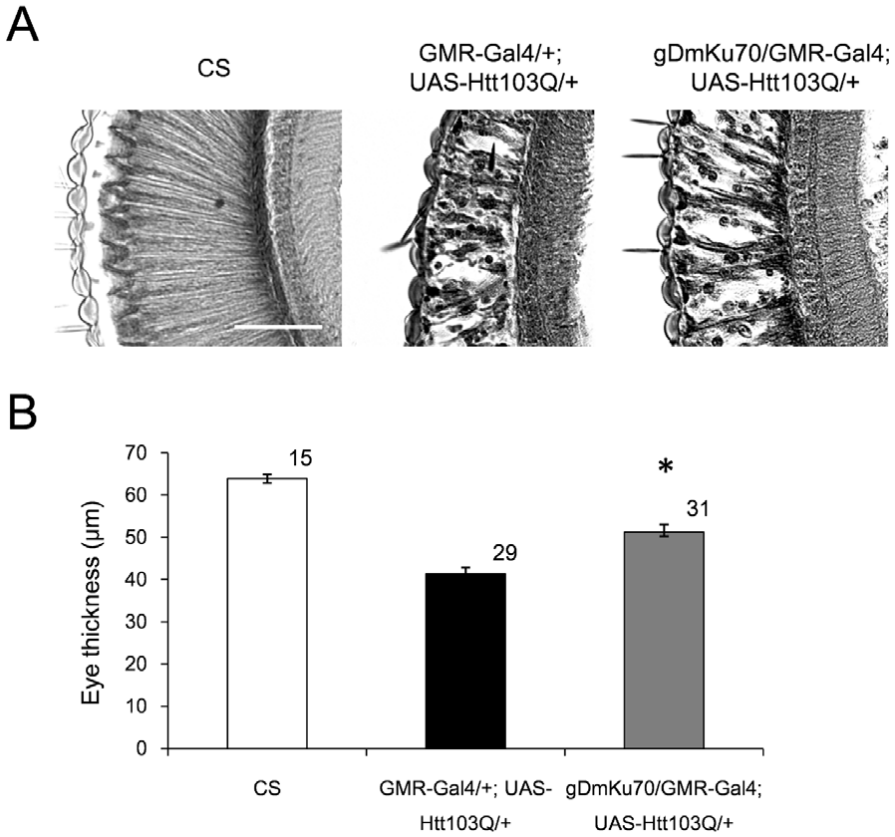
Drosophila homologue of Ku70 (DmKu70) has a protective effect *in vivo* against the toxicity of mutant Htt. (A) Axial sections of the Control Canton-S, GMR-Gal4/+;UAS-Htt103Q/+ and GMR-Gal4/+;gDmKu70/UAS-Htt103Q fly eyes are shown. The vacuolar degeneration was severer than that in Figure 2. In triple transgenic flies generated by mating GMR-Gal4/UAS-Htt103Q flies with UAS-mouse Ku70 flies, Ku70 co-expression partially recovered degeneration. Scale bar, 50 µm. (B) Quantitative analysis of the eye thickness of each transgenic fly. The numbers above each bar indicate the number of counted flies. Mean +/− SE. *: p<0.01, Student's *t*-test. All flies tested were female. Number of flies used for the experiments are indicated on the shoulder of each bar. The genetic background was *w*(CS10) in this figure.

We next tested the effect of Drosophila Ku70 homologue (gDmKu70, a genome rescue construct of Drosophila Ku70) on the mutant Htt-induced eye degeneration to reconfirm the rescuing effect (Figure 2). In this case, we used Htt103Q exon1 peptide (Htt103Q) because slippage-based expansion of polyQ repeats occurred during plasmid construction and we could not control the polyQ length. It might be a reason why eye degeneration was slightly milder in Figure 2 than that in Figure 1. However, beyond the minor difference, Ku70/DmKu70 across species was shown to reduce mutant Htt toxicity in the Drosophila photoreceptor cells.

### Ku70 elongates lifespan of Drosophila HD model

Since genetic interaction between Htt and Ku70/DmKu70 was confirmed in eye degeneration with two independent transgenic lines (Figure 1, 2), we next changed the driver in GAL4-UAS system from GMR to motoneuron-specific OK6. Before this study, we had made various types of neurodegenerative disease fly models using multiple tissue-specific drivers, and had tested their efficiency for screening modifier genes or chemicals (our unpublished observation). Among them, several mutant genes including Htt shorten the lifespan when they are expressed in motor neurons by OK6-GAL4 driver (our unpublished observation). Thus, we decided to use the OK6-GAL4/lifespan model to test the effect of Ku70 on the HD pathology.

At the beginning, we analyzed the life span of genetic background flies used in this study. The life span of the Canton S background flies, *w*(CS10), was shorter than the *w^1118^* background flies in exactly similar culture conditions (Figure S1). The lifespan of *w*(CS10) flies was not affected by Ku70 or OK6-GAL4 transgene (Figure S2).

**Figure 3:**
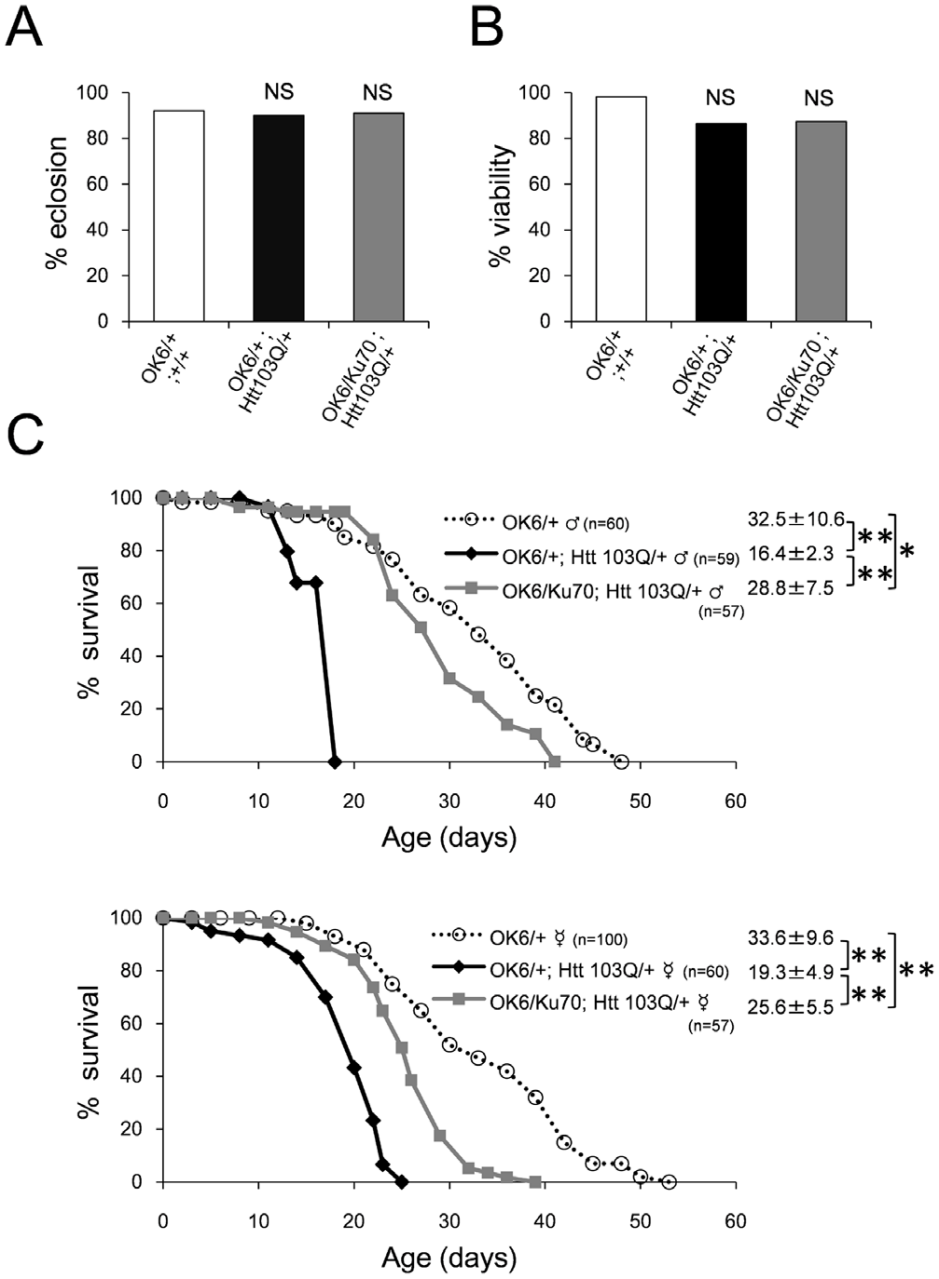
Ku70 reduces *in vivo* toxicity of mutant Htt in Drosophila models. (A) Eclosion rate is not reduced by motor neuron-specific expression of human mutant Htt103Q with OK6-Gal4 driver. Pupae eclosed and became adult flies at a similar percentage in all genotypes (OK6/+; +/+ for control, OK6/+; Htt 103Q/+ for disease model and OK6/Ku70; Htt103Q/+ for recovered flies). χ^2^ statistics was used to test the difference among groups. NS: not significant. (B) Viability is not reduced by motor neuron-specific expression of human mutant Htt103Q with OK6-Gal4 driver (black bar). Viability here means the ratio between balancer-negative flies and balancer-positive flies generated from a single crossing. White bar indicates the ratio between OK6/+;+/+ and CyO/+;+/+, black bar indicates the ratio between OK6/+;Htt103Q/+ and CyO/+;Htt103Q/+, and gray bar indicates the ratio between OK6/Ku70;Htt103Q/+ and CyO/Ku70;Htt103Q/+. χ^2^ statistics was used to test the difference among groups. NS: not significant. (C) Lifespan shortening by OK6-Gal4-driven motor neuron-specific expression of human mutant Htt103Q was rescued by co-expression of Ku70 by the same driver. Survival curves of males (upper panels) and females (lower panels) of control fly (OK6/+), disease model fly (OK6/+; Htt 103Q/+) and rescue fly (OK6/Ku70; Htt 103Q/+) were shown. Number of flies used for the experiments are 60 for OK6/+ males, 59 for OK6/+; Htt 103Q/+ males, 57 for OK6/Ku70; Htt 103Q/+ males, 100 for OK6/+ females, 60 for OK6/+; Htt 103Q/+ females and 57 for OK6/Ku70; Htt 103Q/+ females. The genetic background was *w*(CS10) in this figure. The average lifespan and SD are shown. *p<0.05, **p<0.01, Wilcoxon's test with Bonferroni's correction.

Then, we tested whether expression of human Htt103Q exon1 peptide affects the pupal eclosion rate: percentage of successful eclosion from 100 pupae (Figure 3A) or the viability ratio: ratio between balancer-negative adult flies and balancer-positive adult flies generated from a single crossing (Figure 3B). The pupal eclosion rate reflects the lethality at the pupal developmental stage, while the viability ratio reflects the whole developmental lethality. As described in the legend (Figure 3B), white bar in the graph indicates the ratio of OK6/+;+/+ to CyO/+;+/+, black bar indicates the ratio of OK6/+;Htt103Q/+ to CyO/+;Htt103Q/+, and gray bar indicates the ratio of OK6/Ku70;Htt103Q/+ to CyO/Ku70;Htt103Q/+. From the result of viability ratio (Figure 3B), Htt103Q possessed a weak toxicity during development and Ku70 was not effective for the developmental lethality. Since pupal eclosion rate was not changed by Htt103Q expression (Figure 3A), mutant Htt might be toxic for an early developmental stage of larva.

We next analyzed the lifespan of OK6/+;+/+, OK6/+;Htt103Q/+, and OK6/Ku70;Htt103Q/+. We found obvious lifespan shortening by OK6-Gal4-mediated expression of Htt103Q in motor neurons both in male and female flies (Figure 3C, upper and lower panels). Co-expression of Ku70 clearly recovered the lifespan shortening by Htt103Q in male and female flies, although the recovery did not reach to the level of normal flies (Figure 3C). Co-expression of Ku70 in motor neurons did not affect Htt103Q expression level in quantitative RT-PCR (Figure S3) as in western blot for eye expression (Figure 1E). To exclude the possibility of the transgene insertion effect, we tested another independent transgenic line generated by injection of the UAS-Ku70 vector to another egg. As expected, Ku70 rescued the lifespan shortening by OK6-Gal4-mediated expression of Htt103Q in motor neurons (Figure S4).

### Ku70 reduction affects the lifespan of HD model fly

**Figure 4:**
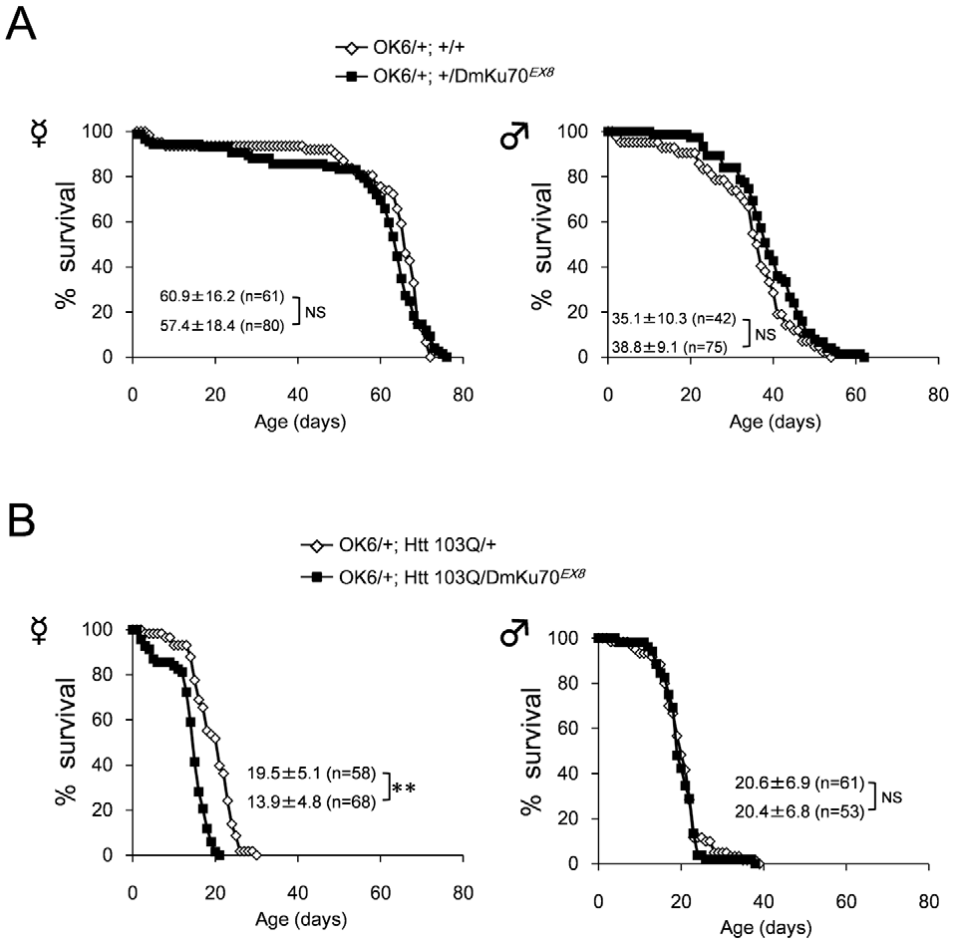
Reduction of DmKu70 enhances *in vivo* toxicity of mutant Htt in Drosophila models. (A) Lifespan is not shortened by null mutation of endogenous DmKu70 in driver (OK6-Gal4/+) control background. The survival curve of heterozygous DmKu70 null mutant (filled square: OK6-Gal4/+; DmKu70^EX8^) was similar to that of DmKu70 wt (open diamond:OK6-Gal4/+; DmKu70^+/+^). (B) Lifespan is shortened by null mutation of endogenous DmKu70 in female Htt model background (OK6-Gal4/+; UAS-Htt103Q/+). The survival of heterozygous DmKu70 null mutant (black square: OK6-Gal4/+; UAS-Htt103Q/+; DmKu70^EX8/+^) was shorter than wt (open diamond: OK6-Gal4/+; UAS-Htt103Q/+; DmKu70^+/+^). Number of flies used for the experiments are 61 for OK6/+ females, 80 for OK6-Gal4/+; DmKu70^EX8/+^ females, 58 for OK6-Gal4/+; UAS-Htt103Q/+ females, 68 for OK6-Gal4/+; UAS-Htt103Q/+; DmKu70^EX8/+^ females, 42 for OK6/+ males, 75 for OK6-Gal4/+; DmKu70^EX8/+^ males, 61 for OK6-Gal4/+; UAS-Htt103Q/+ males and 53 for OK6-Gal4/+; UAS-Htt103Q/+; DmKu70^EX8/+^ males. The genetic background for female flies was *w^1118^* derived from Bloomington Drosophila Stock Center. For male flies, the 2nd chromosome was *w*(CS10) and the 3rd chromosome was *w^1118^* and the X chromosome was the mixture of *w*(CS10) and *w^1118^*. The average lifespan and SD are shown. **p<0.01, Wilcoxon's test.

We tested the effect of Ku70 null mutation on the lifespan of HD model flies. Heterozygous null mutation of Drosophila Ku70 gene (DmKu70^EX8^) did not shorten the lifespan of genetic background flies (Figure 4A). When it was crossed with the HD model flies, the heterozygous Ku70 null mutation accelerated the lifespan shortening by OK6-Gal4-mediated expression of Htt103Q in female flies (Figure 4B). The effect was not large although the difference between the flies was confirmed statistically. The acceleration was not confirmed in male flies (Figure 4B).

Therefore, we next tried to test the effect of homozygous null mutation of DmKu70 on the lifespan of HD model flies. However, it was impossible because the homozygous null mutant flies were embryonic lethal. Instead, we tested the effect of compound heterozygous null mutation of Ku70 by using DmKu70^7B2^. Unexpectedly, we observed that the effect of transheterozygous mutation was smaller than that of single DmKu70^EX8^ mutation. We tested all possibilities that might cause such a result. Finally, we found that the lifespan of background flies for DmKu70^7B2^ was artificially elongated (data not shown) and that the elongation led to the unexpected result in the transheterozygous mutation. Consequently, we could not test the effect by compound heterozygous null mutation of DmKu70^EX8^/DmKu70^7B2^.

### Ku70 reduction accelerates locomotion disability of HD model fly

**Figure 5:**
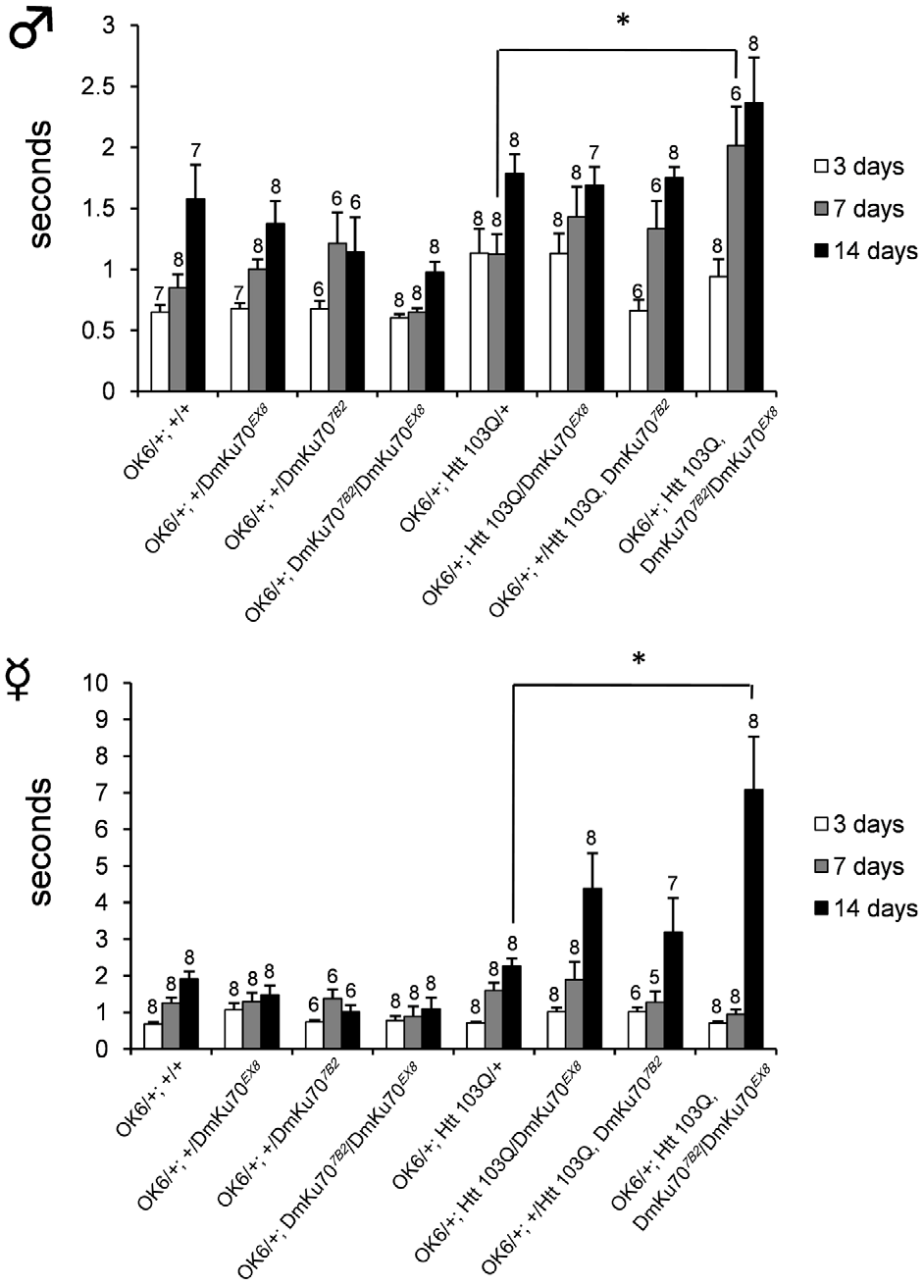
Drosophila Ku70 mutation accelerates locomotion disability of mutant Htt transgenic flies. Startle-induced negative geotactic response (time to reach to 5 cm high) was investigated. Control, heterozygous DmKu70 mutants, transheterozygous mutants, and their Htt103Q expressing flies were tested at 3, 7 and 14 days after eclosion. Htt103Q expressers and non-expressers were compared separately. Significant difference was observed between the HD background and its compound heterozygous mutant both in male and female flies. The numbers of individual fly used for the analysis (N) are indicated above each bar. Experiments were performed three times with each fly, thus the actual “ n ” for statistical analysis was three times of the fly number (N). We employed ANOVA and posthoc Dunnett's test to compare them. *: p<0.05.

To address the effect of Ku70 reduction on HD phenotype, we changed the way and tested whether Ku70 null mutation affects the locomotion ability of Drosophila HD model (Figure 5) using startle-induced negative geotactic response. We put a single fly in to a test column, dropped it to the bottom by knocking the column, and measured the time for fly to reach to 5 cm. By this method, we evaluated locomotion ability of flies at 3, 7, and 14 days after eclosion. In this case, we used transheterozygous null mutation of DmKu70^EX8^/DmKu70^7B2^, because both background did not affect locomotion ability. Although there was no remarkable difference in the antigravity locomotion between OK6/+ control flies and Htt103Q expressing flies, we found Ku70 mutation accelerates the age-dependent decline of locomotion ability in Htt103Q expressing flies, specifically (Figure 5). Interestingly, the acceleration was more prominent in female flies.

Furthermore, we used RNAi-mediated knock down (KD) of Ku70 to confirm the relationship between Ku70 reduction and mutant Htt-dependent phenotype. The result showed that Ku70-KD induced locomotion disability of female HD model flies at 14 days but not of normal flies (Figure S5), supporting that endogenous DmKu70 has a protective effect against the HD pathology.

## Discussion

In this study, we showed that Ku70 alleviates the HD phenotypes in Drosophila models. We also showed that functional deficiency of Ku70 accelerates the lifespan shortening and locomotion disability of HD model flies. These results were consistent with our previous observation that Ku70 remarkably elongates the lifespan of R6/2 mice, the severest mouse model of HD [27], and strongly support the significance of Ku70 as a disease mediator/modifier gene and a therapeutic target.

Mutant Htt reduces DNA-PK activity and impairs the DNA repair function of Ku70 *in vitro* [27]. In the double transgenic mice generated from R6/2 and Ku70 transgenic mice, Ku70 reduces DNA damage of striatal neurons [27]. These findings in our previous study provide the idea that the functional defect of Ku70 underlies the HD pathology. However, it is also possible to speculate that Ku70 blocks mutant Htt to prevent interaction between mutant Htt and other target molecules and/or to inhibit aggregation of mutant Htt. The two hypotheses are inseparable and just like two sides of a coin. If we focus DNA repair, the two stories are identical. If we consider certain pathology other than DNA repair, the blocking effect might be more important.

With Drosophila, we could not directly evaluate DSBs in a few motor neurons. It is because there is no fly orthologue of mammalian H2AX, a direct indicator of DSB, and there is no alternative method to detect DSB in neurons of Drosophila. However, results in this study are basically consistent with our previous observation that impairment of DSB repair is a critical component in the HD pathology [26], [27] and with previous reports showing DNA damage signal activation in HD cell models [26], [34].

The concept of the linkage between DNA damage repair and neurodegeneration is further supported by multiple autosomal recessive cerebellar atrophies, in which mutations of DNA repair genes such as AOA1/EAOH [35], AOA2 [36], and SCAN1 [37], [38] cause neuronal dysfunction and cell death. Therefore, impairment of DNA damage repair could be considered as a common pathological component across categories of neurodegenerative disorder.

In conclusion, our results further support that Ku70 is a critical regulatory factor of Htt toxicity and a candidate for therapeutic target of HD. This study has provided us a rationale to proceed to the next step for translational approaches with Ku70 such as using viral vectors expressing Ku70 or low molecular weight chemicals against DSB in HD.

## Materials and Methods

### Western blot analysis

Western blot analysis was performed as described previously [26]. Briefly, 50 female fly heads were dissolved in 100 µl of 62.5 mM Tris/HCl, pH 6.8, 2% (w/v) SDS, 2.5% (v/v) 2-mercaptoethanol, 5% (v/v) glycerin, and 0.0025% (w/v) bromophenol blue. Samples were separated by SDS-PAGE, transferred onto Immobilon-P Transfer Membrane (Millipore) through a semidry method, blocked by 5% milk in TBS with Tween 20 (TBST) (10 mM Tris/Cl, pH 8.0, 150 mM NaCl, 0.05% Tween 20). The filters were incubated with each primary antibody for overnight at 4°C. Primary antibodies used were; mouse anti-polyglutamine (1∶2000, 1C2, Milipore), mouse anti-Ku70 (1∶500, N3H10, Kamiya Biomedical, Seattle) and mouse anti-actin (1∶2000, C4, Chemicon). The filters were then incubated with horseradish peroxidase (HRP)-conjugated second antibody at a 1∶3000 dilution for 1 h at room temperature in 5% milk/TBST and visualized by Enhanced Chemiluminescence western blot detection system (Amersham Biosciences, GE Health Care Biosciences, Hong Kong).

### Fly stocks and rearing conditions

Canton S, *w*(CS10) and *w^1118^* (Bloomington Drosophila Stock Center) were genetic backgrounds used for this study. All flies were raised and maintained on a corn-meal medium at 25°C and 60% humidity in a 12∶12 hr light-dark cycle. GMR-Gal4 is P{longGMR-GAL4}3 and *GMR-Huntingtin^120Q^* line [40] were obtained from Bloomington Drosophila stock center. UAS-mouse Ku70 transgenic flies possessing a 1.8 kb full length cDNA of AK143570 RIKEN clone were generated from a *w*(CS10) parental strain. *Ku70^EX8^* and *Ku70^7B2^* are deletion mutants of the *Drosophila Ku70* gene reported previously [39]. Genomic DmKu70 transgenic fly (*gDmKu70*) was also reported previously [33]. *UAS-Htt103Q* fly was made by P-element-mediated germline transformation. pUAS-Htt103Q plasmid was constructed by subcloning human *HD* exon1 cDNA digested from pTL1HA3-HD90Q with EcoRI and NotI, into pUAST vector. pTL1HA3-HD90Q was made by replacing the insert of pTL1HA3-HD51Q (pTL1-CAG51) [41]. *w; +/+; UAS-Htt103Q* or *w; gDmKu70; UAS-Htt103Q* virgin females were crossed with an eye specific driver GMR-Gal4 transgenic males, and flies *w; GMR-Gal4/+; UAS-Htt103Q/+* (disease model) and *w; GMR-Gal4/gDmKu70; UAS-Htt103Q/+* (recovery flies) were generated.

To generate Ku70 RNAi fly, KK110409, in which an RNAi construct has been inserted into 2nd chromosome, was purchased from of Vienna Drosophila RNAi Center (VDRC). The virgin female flies, *w*/*w*; RNAi/RNAi; +/+ were crossed with *w*/Y; +/+; *TM3 Ser*/*sb^1^* whose genetic background is *w*(CS10). The resultant F1 virgin females, *w*/*w*; RNAi/+; *TM3 Ser*/+, were crossed with *w*/Y; *CyO*/*sp*; Htt 103Q/Htt 103Q males (*w*(CS10) background). The F2 flies, *w*/Y; *CyO*/RNAi; *TM3 Ser*/Htt 103Q and *w*/Y; *CyO*/+; *TM3 Ser*/Htt 103Q can be distinguished by their eye colors. Each males were crossed with OK6-Gal4 (*w*(CS10) background) virgin females. The final flies were *w*/*w* (or Y); OK6/RNAi; Htt 103Q/+ and *w*/*w* (or Y); OK6/+; Htt 103Q/+.

We thank Dr. Dena Johnson-Schlitz and Dr. William Engels (University of Wisconsin) for gDmKu70 transgenic and *Ku70^EX8^* and *Ku70^7B2^* mutant flies, and Mrs. Tayoko Tajima for her excellent technical support.

### Fly lifespan and viability

The fly lifespan was analyzed as described [42]. The strains used for the analysis were backcrossed more than three times to possess the same genetic background. In a vial, 1 to 20 flies were reared carefully to keep a good condition. Viability (the ratio between balancer-negative adult flies and balancer-positive adult flies generated from a single crossing) was calculated by counting the number of eclosed adult flies of each genotype using balancer phenotype. The lifespan of Canton S and *w^1118^* was different as shown in Figure S1, while the lifespan of a genetic background was not affected by crossing Ku70 or OK6-GAL4 transgenic fly (Figure S4). χ^2^ statistics was used to test the difference of viability among groups.

### Fly eclosion rate

One hundred larvae at 3rd instar climbing on the wall were collected carefully into a food vial. All the larvae pupate in a few hours after collection. We counted the number of eclosed adult fly until 1 week. The eclosion rate of control flies was almost similar among various genotypes (data not shown). χ^2^ statistics was used to test the difference of eclosion among groups.

### Fly eye morphology

Proboscis were removed from heads of adult female flies and fixed in carnoy's solution (ethanol: chloroform: acetate = 6∶3(1) for 5(hours at 4(C, then dehydrated in serial dilutions of ethanol and embedded in paraffin (pathoprep546, m.p. 54(56(C, Wako). Six((m sections were stained with hemotoxylin-eosin (Merk) after re-hydration. The sections were observed with a microscope and analyzed using computer software, Metamorph (Molecular Devices, Toronto, Canada).

### Startle-induced negative geotactic response

A single fly was transferred in to a test column (150 mm in length and 25 mm in diameter) lined with nylon mesh. We tapped the column to drop the fly and recorded time for the fly to reach 5 cm from the bottom by a time watch. When a fly did not reach 5 cm within 10 sec, the time was recorded as 10 sec. The test was repeated 3 times for each fly.

### Quantitative PCR

Human Htt mRNA levels in transgenic flies were assessed by quantitative real-time PCR (qPCR). Ten flies were collected from each line, and total RNA was isolated from their whole bodies using RNAeasy mini (Invitrogen). To eliminate genomic DNA contamination, each sample was treated with DNase I (Promega, Madison, WI) on column. First-strand cDNA was synthesized from 200 ng of total RNA using Superscript II VILO (Invitrogen). For qPCR, SYBER Green assays were performed using the THUNDERBIRD (TOYOBO). The primers for Htt were Htt-Forward, 5′-ATGGCGACCCTGGAAAAGCT-3′ and Htt-Reverse, 5′-CGGTGGCGGCTGTTGCTG-3′. Actin5C used as the internal control was quantified with Actin5C-Forward, 5′-CCGAGCGCGGTTACTCTTT-3′and Actin5C-Reverse, 5′-CAACATAGCACAGCTTCTCCTTGAT-3′. The reactions were analyzed by Applied Biosystems (Foster City, CA) Prism 7500.

## Supporting Information

Figure S1 **Different lifespans between Canton S and** ***w^1118^*** **genetic background flies.** The lifespan was different among Canton S homozygous, *w^1118^* homozygous, and compound heterozygous background flies.
(TIF)

Figure S2 **The lifespan of the Canton S background flies was not affected by OK6 or Ku70 transgene.** (A) Lifespan was not different between genetic controls and Ku70 overexpression fly. OK6/+ (w/Y; OK6-Gal4/+; +/+), Ku70/+; Htt 103Q/+ (w/Y; UAS-mKu70/+; UAS-Htt 103Q/+) and OK6/Ku70 (w/Y; OK6-Gal4/UAS-mKu70; +/+). All flies tested were male. (B) Eclosion rate was not different between genetic controls and Ku70 overexpression fly. The genotypes were, Canton-S (wild type), OK6 (w; OK6-Gal4/+; +/+), Ku70-Htt 103Q (w; UAS-mKu70/+; UAS-Htt 103Q/+), OK6-Ku70 (w; OK6-Gal4/UAS-mKu70; +/+), OK6-Htt 103Q (w; OK6-Gal4/+; UAS-Htt 103Q/+) and OK6-Htt 103Q-Ku70 (w; OK6-Gal4/UAS-mKu70; UAS-Htt 103Q/+).
(TIF)

Figure S3 **Htt103Q expression was not affected by UAS-Ku70 transgene.** To exclude deprivation of GAL4 transcription factor from UAS-Htt103Q by UAS-Ku70 underlies amelioration of the phenotype, we performed RT-PCR to test expression level of Htt103Q in different transgenic flies. The expression level of Htt103Q was not changed by UAS-Ku70 transgene (n = 3, Student's *t*-test). Although recovery was larger in male flies (Figure 3C), Htt was higher in male than female of Ku70 expressing flies.
(TIF)

Figure S4 **Ku70 elongates the lifespan of an independent line of Htt103Q-expressing flies.** To exclude the insertion effect of the Ku70 transgene, we tested another transgenic line generated by independent injection of the UAS-Ku70 vector. Consistently with the result in Figure 3C, we observed that Ku70 rescued the lifespan shortening by Htt103Q.
(TIF)

Figure S5 **Knock down of Drosophila Ku70 accelerates locomotion disability of mutant Htt transgenic flies.** Startle-induced negative geotactic response was used to assess locomotion ability of flies, and time to reach to 5 cm high was counted. To test the effect of DmKu70 knock down on mutant Htt expressing flies, we compared the time between Htt103Q transgenic flies and DmKu70-KD/Htt103Q transgenic flies at 3, 7 and 14 days after eclosion. We found acceleration of locomotion disability by DmKu70-KD in female flies. We employed ANOVA and posthoc Dunnett's test to compare them. *p<0.05.
(TIF)
