# Correction: Ku70 Alleviates Neurodegeneration in Drosophila Models of Huntington's Disease

**DOI:** 10.1371/annotation/e436994f-3b22-4b35-bd29-49992f64a584

**Published:** 2012-01-18

**Authors:** Takuya Tamura, Masaki Sone, Takeshi Iwatsubo, Kazuhiko Tagawa, Erich E. Wanker, Hitoshi Okazawa

There is an error in the funding statement. The correct funding statement is, "This work was supported by grants to H.O. from Japan Science Technology Agency (CREST), from The Ministry of Education, Culture, Sports, Science and Technology of Japan (Grant-in-Aid for Scientific Research on Innovative Areas "Foundation of Synapse and Neurocircuit Pathology" and Strategic Research Program of Brain Sciences), and from Tokyo Medical and Dental University (Joint Usage/Research Program of Medical Research Institute). Financial support to E.E.W. was provided by National Genome Research Network (NGFN) grants and DFG Collaborative Research Centre grants (SFB577, SFB740, SFB618). The funders had no role in study design, data collection and analysis, decision to publish, or preparation of the manuscript." 

