# Supplementary figures and images for: Ku70 Alleviates Neurodegeneration in Drosophila Models of Huntington's Disease
Source: PLoS One. 2011 Nov 7;6(11):e27408. doi: 10.1371/journal.pone.0027408 (PMC3210167; doi:10.1371/journal.pone.0027408)

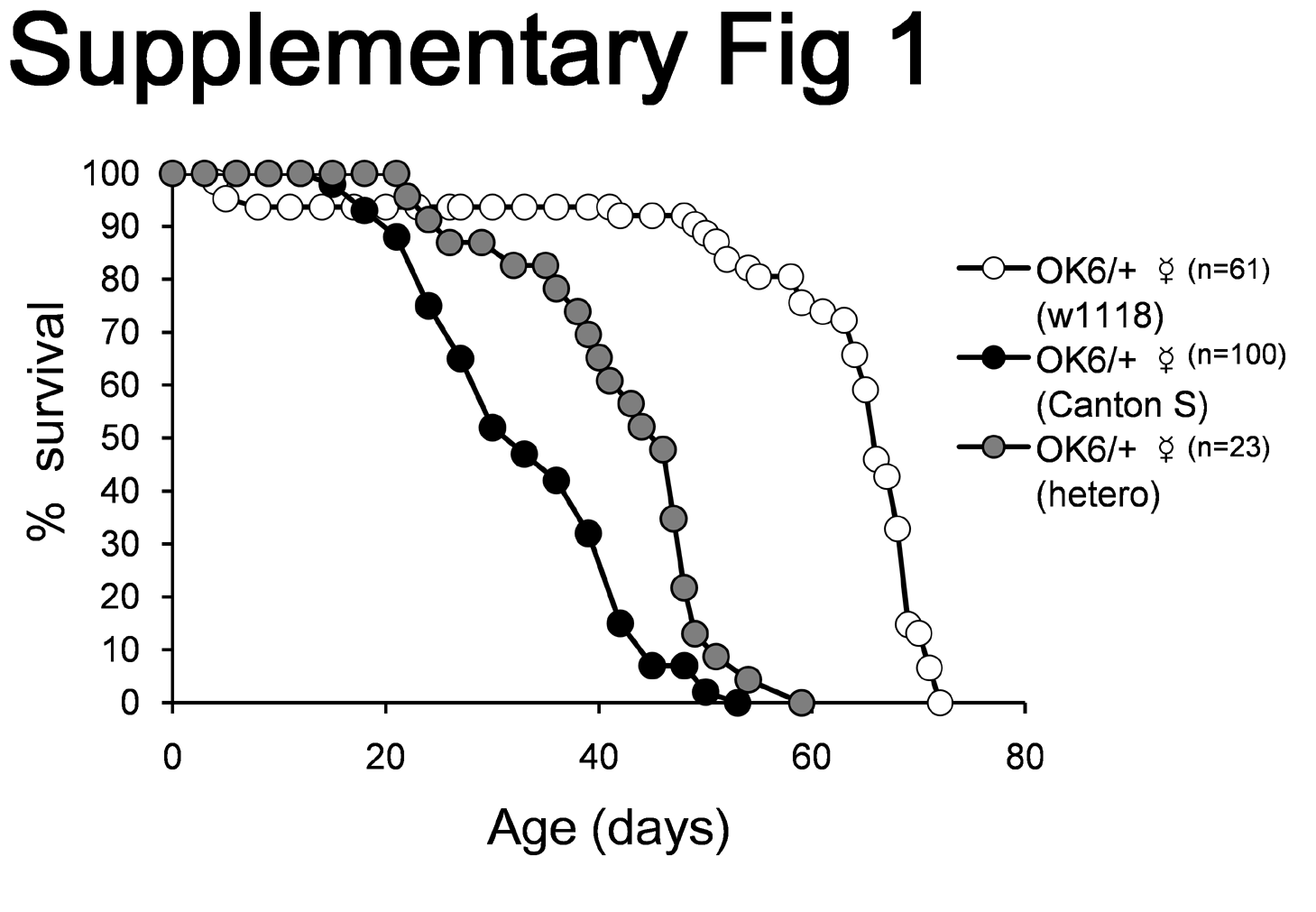

Supplement: Figure S1 — Different lifespans between Canton S and w1118 genetic background flies. The lifespan was different among Canton S homozygous, w1118 homozygous, and compound heterozygous background flies. (TIF) [file pone.0027408.s001.tif]

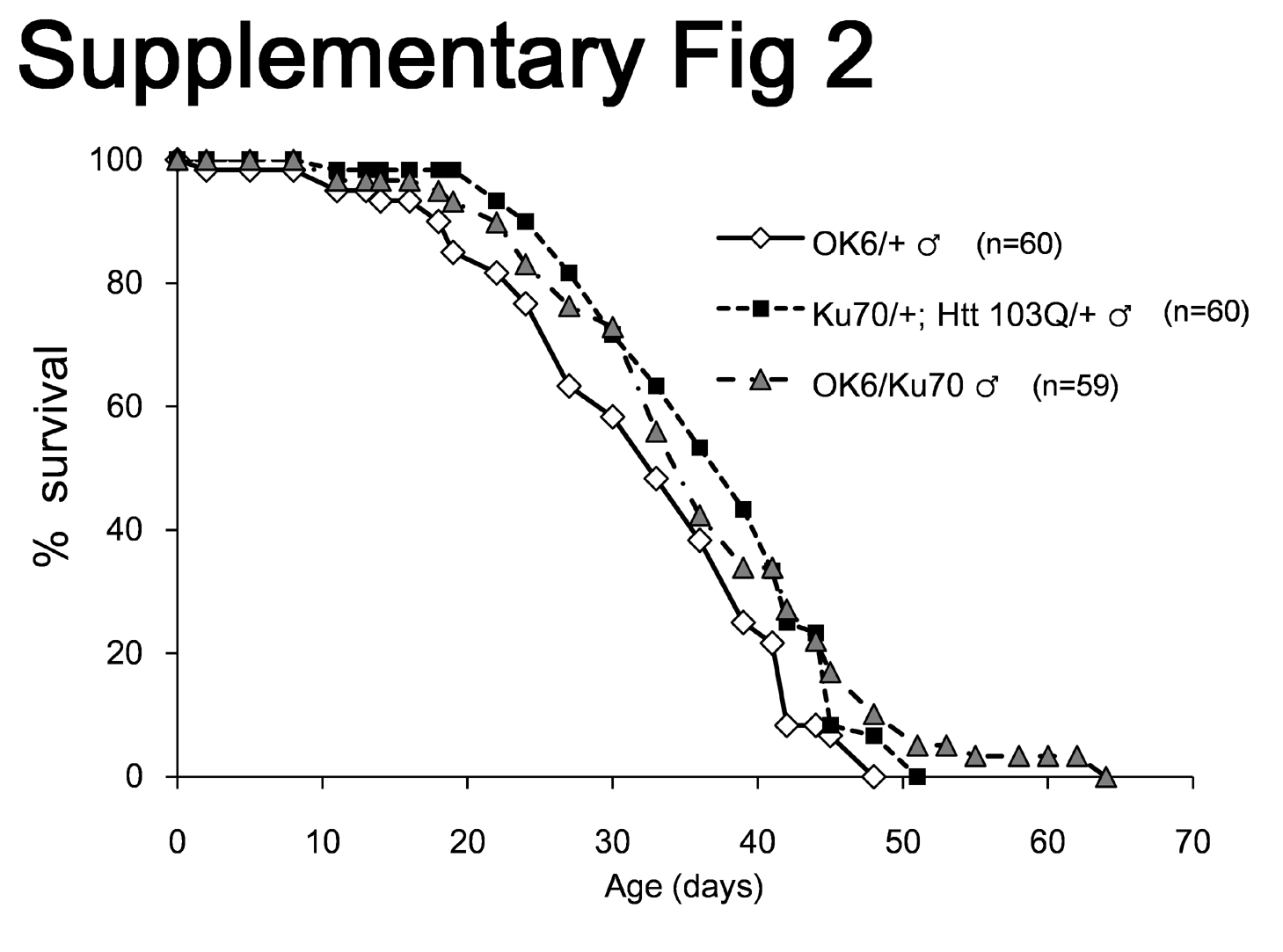

Supplement: Figure S2 — The lifespan of the Canton S background flies was not affected by OK6 or Ku70 transgene. (A) Lifespan was not different between genetic controls and Ku70 overexpression fly. OK6/+ (w/Y; OK6-Gal4/+; +/+), Ku70/+; Htt 103Q/+ (w/Y; UAS-mKu70/+; UAS-Htt 103Q/+) and OK6/Ku70 (w/Y; OK6-Gal4/UAS-mKu70; +/+). All flies tested were male. (B) Eclosion rate was not different between genetic controls and Ku70 overexpression fly. The genotypes were, Canton-S (wild type), OK6 (w; OK6-Gal4/+; +/+), Ku70-Htt 103Q (w; UAS-mKu70/+; UAS-Htt 103Q/+), OK6-Ku70 (w; OK6-Gal4/UAS-mKu70; +/+), OK6-Htt 103Q (w; OK6-Gal4/+; UAS-Htt 103Q/+) and OK6-Htt 103Q-Ku70 (w; OK6-Gal4/UAS-mKu70; UAS-Htt 103Q/+). (TIF) [file pone.0027408.s002.tif]

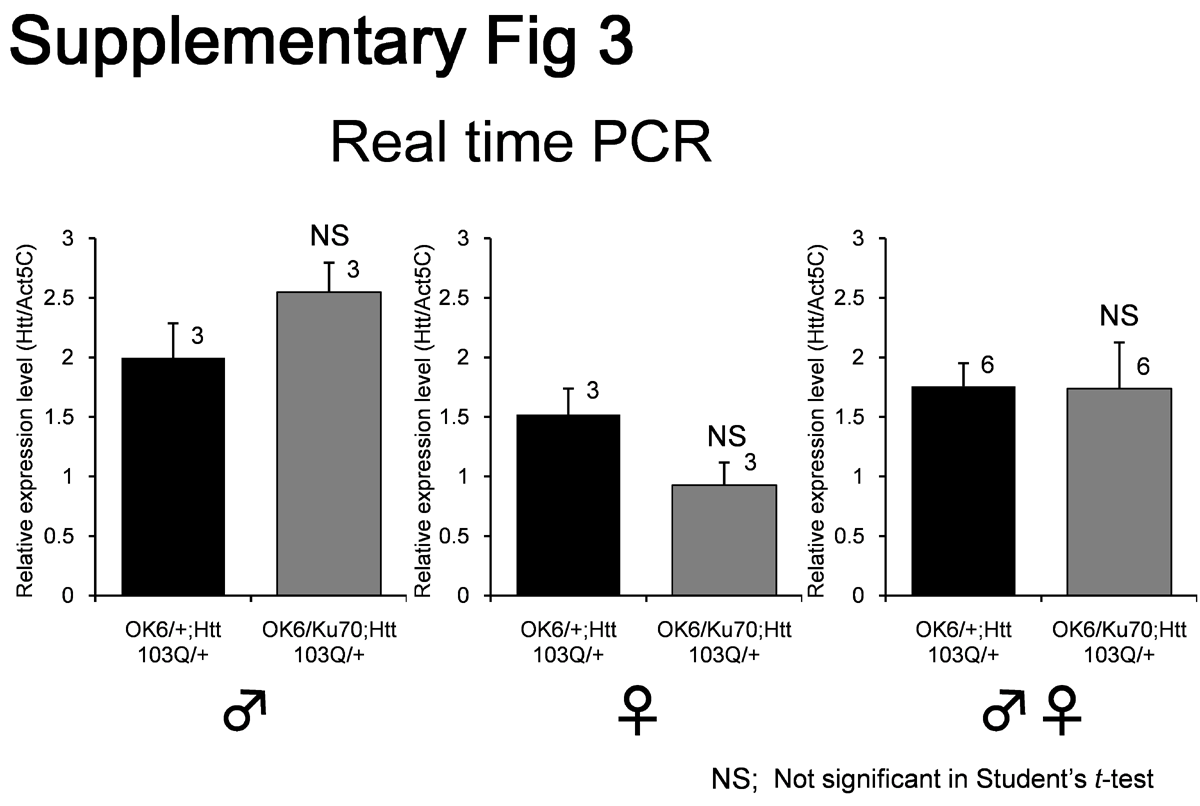

Supplement: Figure S3 — Htt103Q expression was not affected by UAS-Ku70 transgene. To exclude deprivation of GAL4 transcription factor from UAS-Htt103Q by UAS-Ku70 underlies amelioration of the phenotype, we performed RT-PCR to test expression level of Htt103Q in different transgenic flies. The expression level of Htt103Q was not changed by UAS-Ku70 transgene (n = 3, Student's t-test). Although recovery was larger in male flies (Figure 3C), Htt was higher in male than female of Ku70 expressing flies. (TIF) [file pone.0027408.s003.tif]

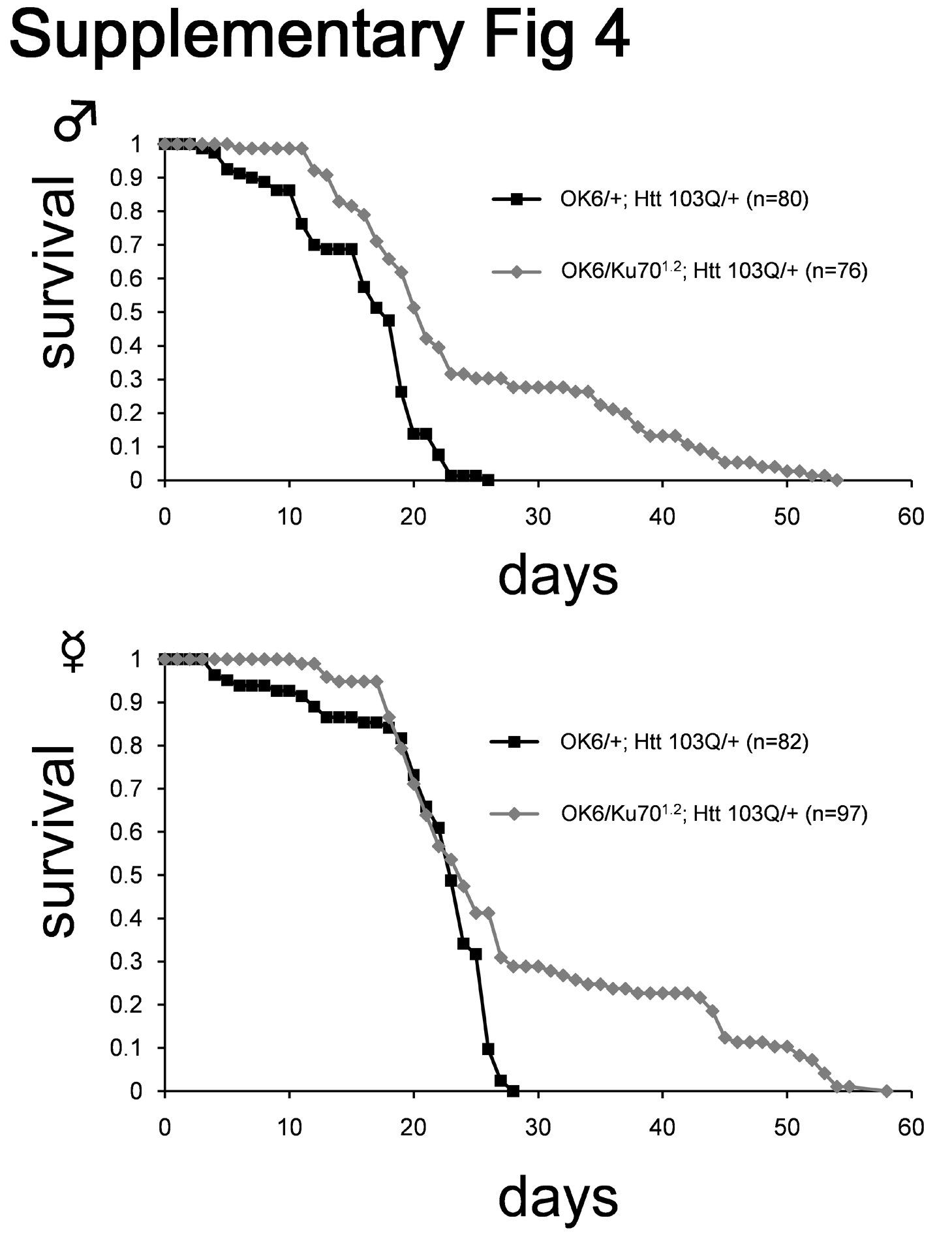

Supplement: Figure S4 — Ku70 elongates the lifespan of an independent line of Htt103Q-expressing flies. To exclude the insertion effect of the Ku70 transgene, we tested another transgenic line generated by independent injection of the UAS-Ku70 vector. Consistently with the result in Figure 3C, we observed that Ku70 rescued the lifespan shortening by Htt103Q. (TIF) [file pone.0027408.s004.tif]

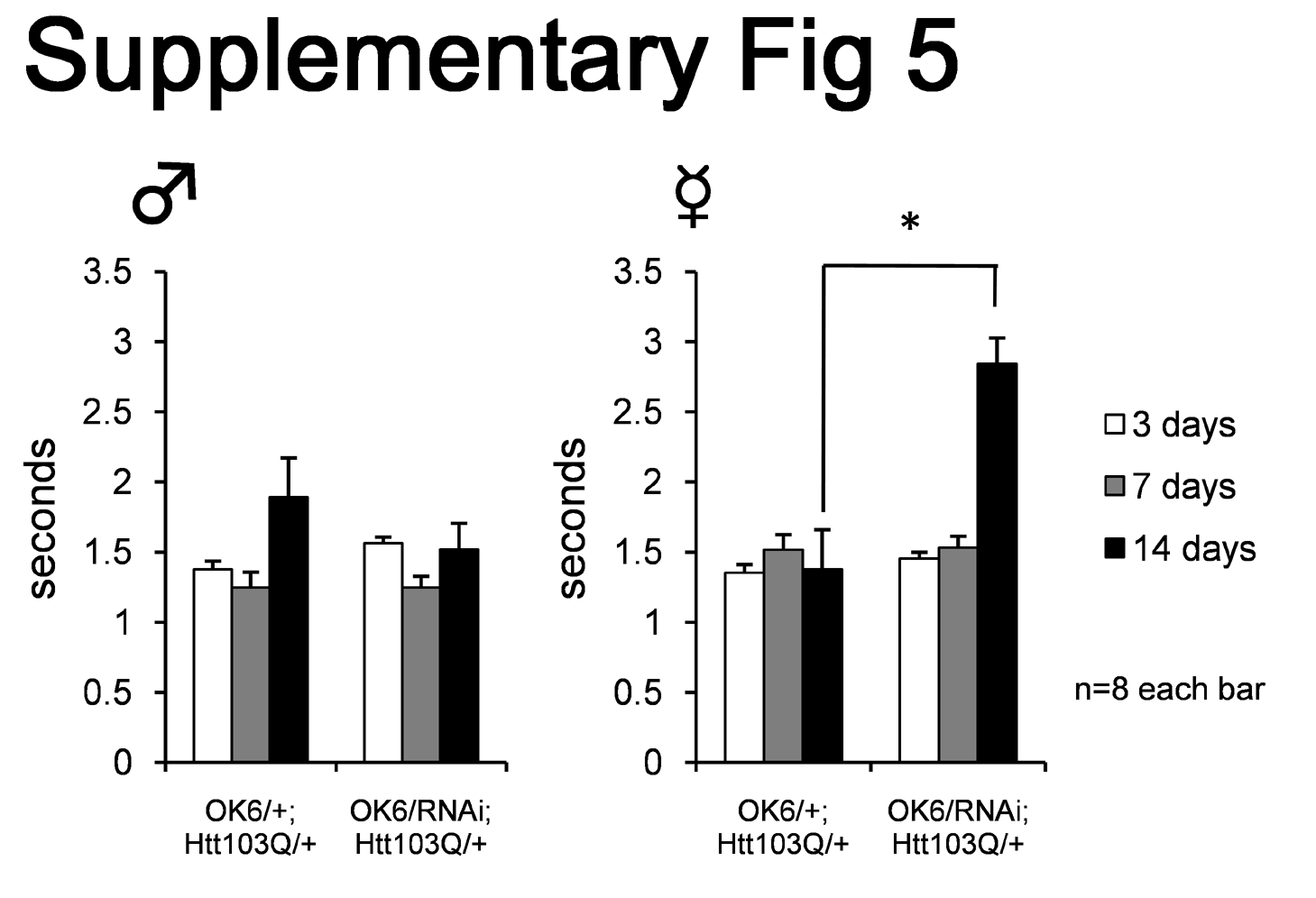

Supplement: Figure S5 — Knock down of Drosophila Ku70 accelerates locomotion disability of mutant Htt transgenic flies. Startle-induced negative geotactic response was used to assess locomotion ability of flies, and time to reach to 5 cm high was counted. To test the effect of DmKu70 knock down on mutant Htt expressing flies, we compared the time between Htt103Q transgenic flies and DmKu70-KD/Htt103Q transgenic flies at 3, 7 and 14 days after eclosion. We found acceleration of locomotion disability by DmKu70-KD in female flies. We employed ANOVA and posthoc Dunnett's test to compare them. *p<0.05. (TIF) [file pone.0027408.s005.tif]
